# First person – Rita Aires and Julia Kramer

**DOI:** 10.1242/dmm.052097

**Published:** 2024-09-27

## Abstract

First Person is a series of interviews with the first authors of a selection of papers published in Disease Models & Mechanisms, helping researchers promote themselves alongside their papers. Rita Aires and Julia Kramer are co-first authors on ‘ Axolotl mandible regeneration occurs through mechanical gap closure and a shared regenerative program with the limb’, published in DMM. Rita is a postdoctoral researcher in the lab of Tatiana Sandoval-Guzmán at the Center for Healthy Aging (University Hospital Carl Gustav Carus, Technische Universität Dresden, Dresden, Germany). She is passionate about development and regeneration, and in establishing new animal models for their study. Julia is a Specialist in oral and maxillofacial surgery in the lab of Günter Lauer at the Clinic of Oral and Maxillofacial Surgery (University Hospital Carl Gustav Carus Dresden, Technische Universität Dresden, Dresden, Germany). Her focus is on the development, regeneration and oncology of the head and neck region.


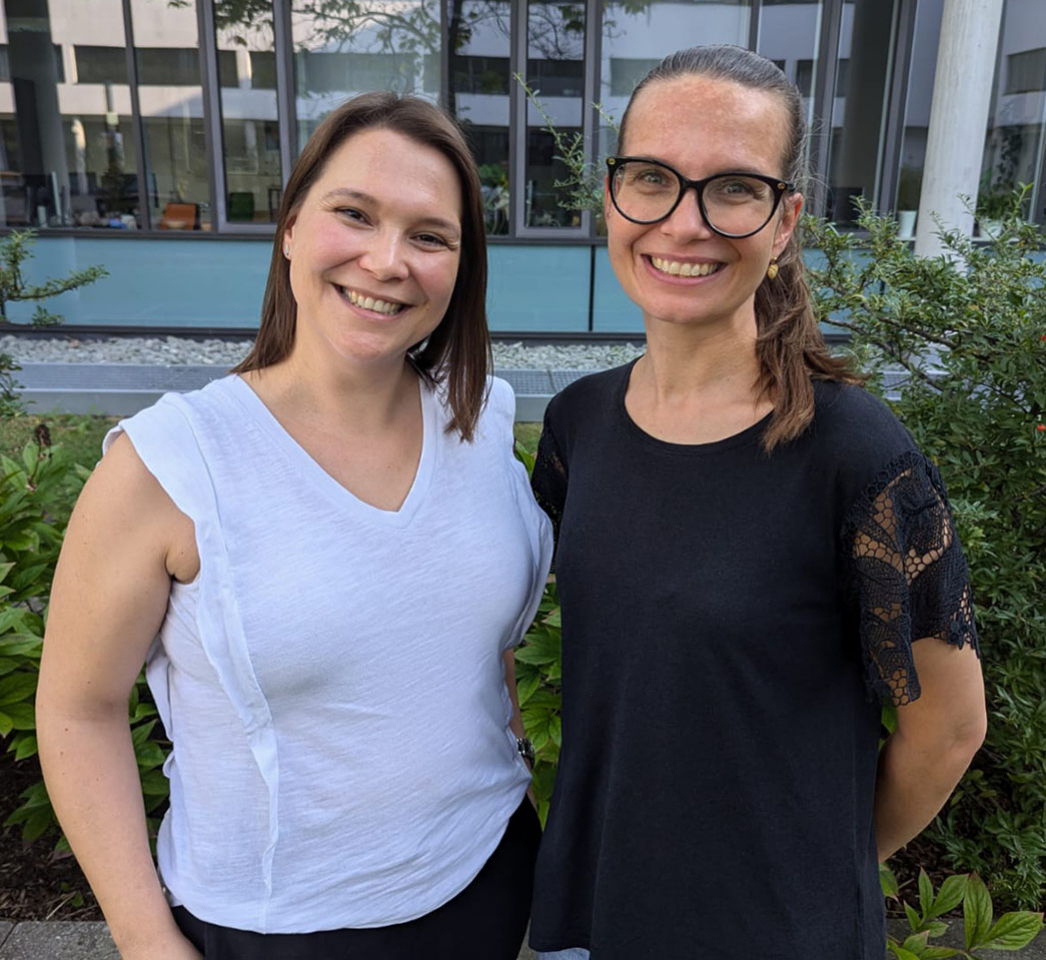


**Rita Aires (left) and Julia Kramer (right)**

**Who or what inspired you to become a scientist?**

**R.A:** I don't think I can pinpoint a moment or person that inspired me to become a scientist. For me, it was a natural progression of wanting to know *more* about how things worked, until I finally realized that there was a whole career for people dedicated to that! One day, during my bachelors, I saw a chicken embryo in the egg for the first time and my fate was sealed: I became an evolutionary and developmental biologist.

**J.K.:** When I started studying medicine, the outlook for prospective physicians was still very difficult. Being scientifically engaged was the requirement for a certain specialization or to work in a university hospital – so, to be honest, that was my initial motivation for starting research. But the exciting work with the incredible model animal axolotl and collaborations with scientists from all over the world as part of my doctoral thesis at the Institute of Anatomy/Developmental Biology awakened a real love at second sight!

[The axolotl] is able to regenerate many organs and structures, including full limbs

**What is the main question or challenge in disease biology you are addressing in this paper? How did you go about investigating your question or challenge?**

**J.K.:** Humans have a limited regeneration potential, and one such limitation is that we cannot regenerate extensive defects in our jaws. Unfortunately, human jaws – especially the lower jaw – are also very prone to damage, either as a consequence of trauma or of certain types of cancer that may require large amounts of it to be taken out. Sadly, extensive lower jaw loss can severely affect the quality of life of patients, given that they are essential for communication and mastication, and are aesthetically very important. So, we asked ourselves how species that do have higher regenerative abilities deal with lower jaw defects, since this knowledge can inspire us in the direction of new and, perhaps, unexpected, future treatments to heal human jaw defects.

**R.A.:** That's why we decided to focus on the axolotl – it is considered to be the master regenerator among vertebrates. This animal is able to regenerate many organs and structures, including full limbs. So, not only were we able to characterize, for the first time, how the axolotl can regenerate extensive mandible defects, but we could also take advantage of the fact that limb regeneration is so well studied in this model and were able to compare the molecular programs used during the regeneration of both structures.

**How would you explain the main findings of your paper to non-scientific family and friends?**

**R.A.:** Us humans cannot regenerate our jaws or our limbs. However, there are some animals, like the axolotl, which can do it – and can even regenerate full limbs and large gaps in their jaws. In this work, we looked into how the axolotl regenerates its lower jaw after creating a large defect in it. We realized that they start by first making the defect smaller. Then they use a set of molecular instructions to regenerate their jaw, which are similar to those they use when regenerating their limbs. This shows that axolotls have a shared molecular program to regenerate their jaws and limbs, but also that the way the axolotl regenerates each structure has its own quirks, such as the lower jaw bones are moving together to make the defect smaller during jaw regeneration.

**J.K.:** As a surgeon, I was particularly fascinated to see how, in the axolotl − without any external intervention and within just a few weeks − jaw fragments find each other again and become aligned in the optimal direction. The defect is then bridged by the formation of tissue that had previously been lost. This is in clear contrast to my professional experience, where mandibular defects pose challenges for patients and clinicians, and require extensive surgery.

**What are the potential implications of these results for disease biology and the possible impact on patients?**

**R.A.:** With this study, we hope to convey the message that, by studying other animal species that can regenerate structures we humans cannot, it is essential to find new therapeutic approaches. By demonstrating that the axolotl has a shared regenerative program for limb and lower jaw, we show that there might be a core set of instructions for axolotl regeneration out there. This could be a starting point to unveil what makes these animals regenerate so competently and, most importantly, what we humans might be missing.

**J.K.:** Also, in recent years, many procedures have been developed to get to the bottom of the causes of certain diseases in the oral and maxillofacial region, and to improve therapeutic options. Perhaps our model can provide new ideas to solve problems with wound-healing disorders in the jaw area through certain factors or cells.

I, personally, appreciate all the work The Company of Biologists has done for the community, especially in providing additional funding opportunities for researchers.

**Figure:**
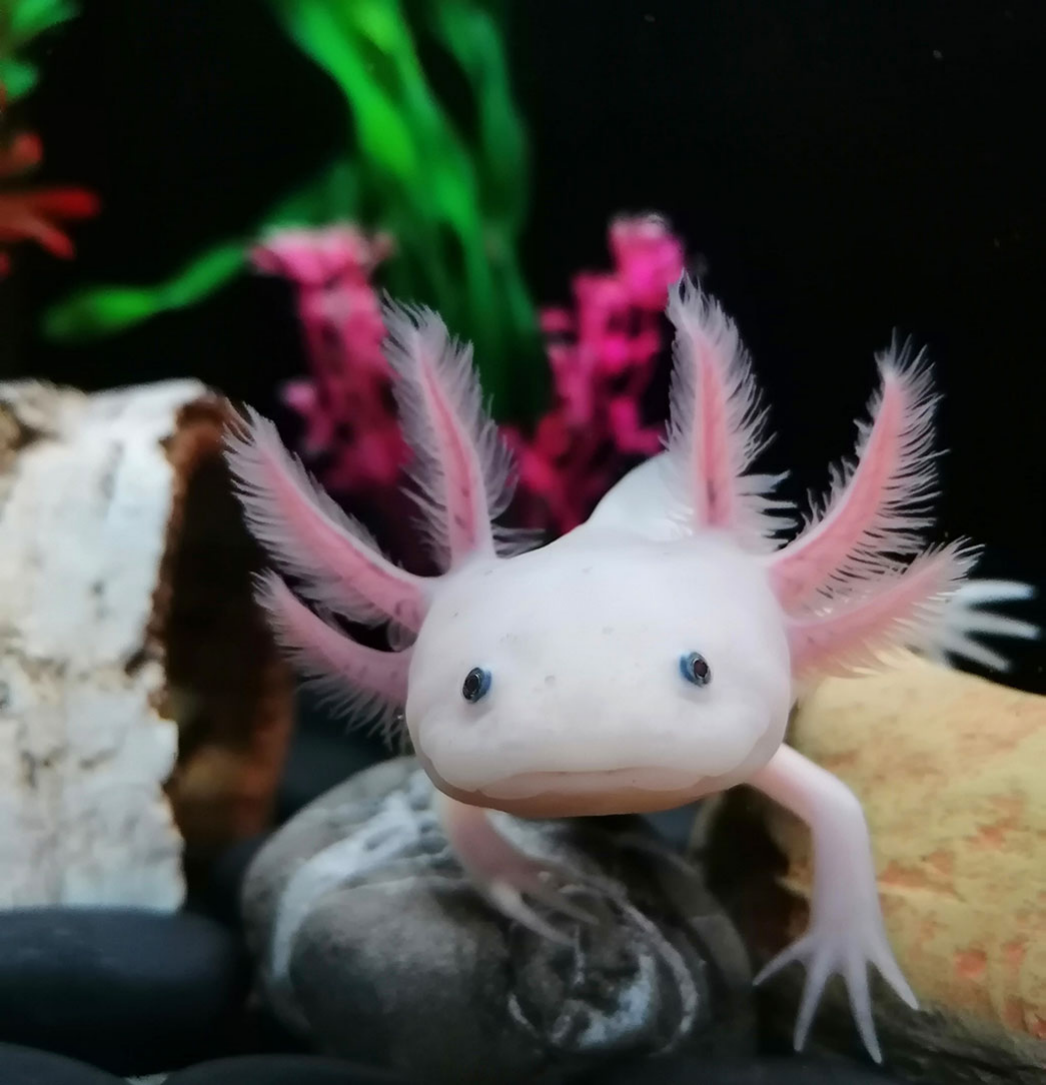
**The axolotl is a master regenerator among vertebrates, and is able to regenerate full limbs and the lower jaw.** Shown here is axolotl Normando. Photo credit: Rita Aires.

**Why did you choose DMM for your paper?**

**R.A.:** Since this work resulted from a collaboration between medical doctors and scientists, we thought that DMM was the right fit for us owing to the journal's great reputation and interest in the mechanisms of human disease by using animal models. Also, I, personally, appreciate all the work The Company of Biologists has done for the community, especially in providing additional funding opportunities for researchers. I was once sponsored to attend an important conference by The Company of Biologists, and I am incredibly grateful!

**J.K.:** Both the title and the scientific profile of the journal were a perfect match for our interdisciplinary project involving biologists and clinicians. Also, the close cooperation of DMM with institutes of the TU Dresden and the support of young scientists by The Company of Biologists, as Rita was able to experience for herself, further encouraged us in our decision.

**Given your current role, what challenges do you face and what changes could improve the professional lives of other scientists in this role?**

**R.A.:** From a postdoc point of view, these − definitely – would be career stability and better salaries. I know too many good scientists, who had to make the hard choice of choosing a more stable job so that they make a living or even start a family, instead of continuing following their passion for research in academia.

**J.K.:** As a physician, it is a particular challenge to find enough time to continuously work on a scientific project in addition to the time-consuming and demanding work with patients − which I do genuinely love. I would like to have more time for research but this is, currently, difficult owing to the tense situation regarding personnel and the heavy workload in hospitals. Without the great collaboration with Rita and the Sandoval-Guzmán lab, this project would not have been possible for me.

**What's next for you?**

**R.A.:** I am currently on the job market looking for a PI position to start my own group, using the axolotl as a model to further study limb and jaw regeneration.

**J.K.:** I am looking forward to expanding my surgical skills even further and to keep up to date with the latest developments to be able to take further career steps in the clinic.

**Tell us something interesting about yourself that wouldn't be on your CV**

**R.A.:** I have two pet axolotls, Normando and Lucky. Normando even features in Fig. 3C of our paper, and you can see him above! He is quite a handsome axolotl, so most pictures I use in my papers or talks are of him.

**J.K.:** When I was only a few months old, a small benign tumor containing parts that resembled small teeth was removed from my face. Even as a child, I found this story both incredibly fascinating and a little scary at the same time. After my studies, I decided to specialize in oral and maxillofacial surgery, with a thesis on the cranial neural crest – that's when I worked with axolotls for the first time. I can't say for sure whether this was just a coincidence or whether it influenced me indirectly.
